# Zerumbone, a Bioactive Sesquiterpene, Ameliorates Diabetes-Induced Retinal Microvascular Damage through Inhibition of Phospho-p38 Mitogen-Activated Protein Kinase and Nuclear Factor-κB Pathways

**DOI:** 10.3390/molecules21121708

**Published:** 2016-12-11

**Authors:** Wayne Young Liu, Thing-Fong Tzeng, I-Min Liu

**Affiliations:** 1 Center for Basic Medical Science, Collage of Health Science, Central Taiwan University of Science and Technology, Taichung 40601, Taiwan; waynedoctor@gmail.com; 2 Department of Urology, Jen-Ai Hospital, Taichung 41625, Taiwan; 3 Department of Pharmacy and Master Program, Collage of Pharmacy and Health Care, Tajen University, Pingtung 90741, Taiwan; d850084@yahoo.com.tw

**Keywords:** diabetic retinopathy, NF-κB, p38 MAPK, zerumbone

## Abstract

Zerumbone ameliorates retinal damage by blocking advanced glycation end products and their receptor system in streptozotocin-diabetic rats. Because of the multiple factors involved in diabetic retinopathy (DR) etiology, the mechanisms of zerumbone that are mainly responsible for its ameliorative effect on DR need to be further clarified. In the present study, zerumbone (20 mg or 40 mg/kg) or fenofibric acid (100 mg/kg) was orally administered to diabetic rats by intragastric gavage once daily for three consecutive months. Zerumbone displayed similar characteristics to fenofibric acid in reducing retinal vascular permeability and leukostasis in diabetic rats. Fundus photographs showed that large retinal vessel diameters were decreased in zerumbone-treated diabetic rats. Zerumbone not only down-regulated the gene expression of retinal angiogenic parameters, but also reduced the expression of inflammatory cytokines and chemokines in the retina of diabetic rats. Moreover, zerumbone reduced the p38 MAPK phosphorylation and abrogated the nuclear translocation of NF-κB p65 in the retina of diabetic rats. In conclusion, treatment of diabetic rats with zerumbone attenuates the severity of retinal inflammation and angiogenesis, via inhibition of p38 MAPK and NF-κB signaling pathways. These benefits of zerumbone for DR appear to be linked to its antihyperglycemic and antihyperlipidemic effects.

## 1. Introduction

Diabetic retinopathy (DR) is known to be the most common microvascular complication of diabetes mellitus (DM). The relative risk of developing DR is higher for type 1 diabetes compared to type 2 [1]. DR is characterized by capillary basement membrane thickening, pericyte and endothelial cell loss, blood-retinal barrier (BRB) breakdown and leakage, acellular capillaries, and neovascularization [2]. Vision loss from DR is a major and leading cause of blindness in adults [3]. There is thus a pressing need for the development of novel and effective therapeutic approaches to halt the progression of DR. The primary pathogenic factor for the progression of DM is hyperglycemia, and a reduction in glycemia has been found to inhibit the development and progression of retinopathy [4]. Unfortunately, tight glycemic control is difficult for many patients with hyperglycemia, so effective supplemental therapies still are needed to inhibit retinopathy.

A number of biochemical pathways have been proposed as potential links between hyperglycemia and DR. There is increasing evidence that hyperglycemia-induced inflammatory processes, including activation of inducible nitric oxide synthase (iNOS) and cyclooxygenase (COX)-2 and production of tumor necrosis factor (TNF)-α, interleukin (IL)-1β and vascular endothelial growth factor (VEGF), have a considerable role in the pathogenesis of DR [5]. These mediators in turn contribute to the upregulation of the adhesion molecules of endothelial cells and leucocytes [5]. Further, leukostasis leads to vascular occlusion, tissue ischemia, edema, loss of neuronal functions, and neuronal cell death [6]. In fact, the increased expression of many inflammatory proteins is regulated at the level of gene transcription through the activation of proinflammatory transcription factors, including nuclear factor-κB (NF-κB) [7]. Due to the involvement of the inflammatory processes of low-grade chronic inflammation in the pathogenesis of DR, inhibiting the inflammatory pathway could be an appealing treatment option for DR in future practices [8].

Zerumbone (1,(2*E*,6*E*,10*E*)-2,6,9,9-tetramethylcycloundeca-2,6,10-trien-1-one) a southeast Asian ginger sesquiterpene, is derived from several plant species of the family Zingiberaceae [9,10]. It is the main component in the rhizome of the edible plant *Zingiber zerumbet* Smith grown in tropical and subtropical areas [11,12], mainly in Southeast Asia. The plant has been shown to possess antinociceptive, anti-inflammatory, antiulcer, antihyperglycemic, and antiplatelet activities [12]. Zerumbone is commonly used as a condiment for food flavoring and has been found to have multiple biomedical properties, such as antiproliferative, antioxidant, anti-inflammatory, and anticancer activities [12,13]. It has also been found that zerumbone could mitigate nutritional steatohepatitis through regulating key genes related to oxidative stress, inflammation and fibrogenesis [14]. Furthermore, the compound is valued for its anti-diabetic properties, which involve antihyperglycemia accompanied by inhibition of hyperglycemia-affected proinflammatory factors, chemokines, or adhesion molecule expression in the kidneys of streptozotocin (STZ)-diabetic rats [15]. In a recent work, zerumbone was found to be beneficial in the amelioration of hyperglycemia-induced retinal damage by the blockade of the advanced glycation end products (AGEs) and their receptor (RAGE) system in STZ-diabetic rats [16]. Zerumbone might therefore be utilized as an adjuvant therapy in the control of diabetic microvascular complications.

Although uncontrolled hyperglycemia-related tissue damage is the primary cause of diabetic microvascular complications, other metabolic abnormalities, such as dyslipidemia, are involved in the progression of DR [17]. Actually, the anti-hyperlipidemic activities induced by zerumbone have been documented in our previous study [18]. Whether the beneficial effects of zerumbone on DR are associated with its plasma lipid lowering activity has yet to be clarified. In the present study, zerumbone was administered in different doses to assess the protective effect of this compound against DR in STZ-diabetic rats, and to evaluate the potential causal mechanisms. Fenofibrate is a common lipid-lowering drug and a potent agonist of the peroxisome proliferator-activated receptor α (PPARα), that has been shown to be effective in reducing the risk of progression of DR, although this effect does not seem to be attributable to changes in lipid profile [19,20]. As such, fenofibric acid, the active metabolite of fenofibrate [20], was used as the positive control in the present study.

## 2. Results

### 2.1. Effects on Body Weights and Plasma Parameters

**Figure 1:**
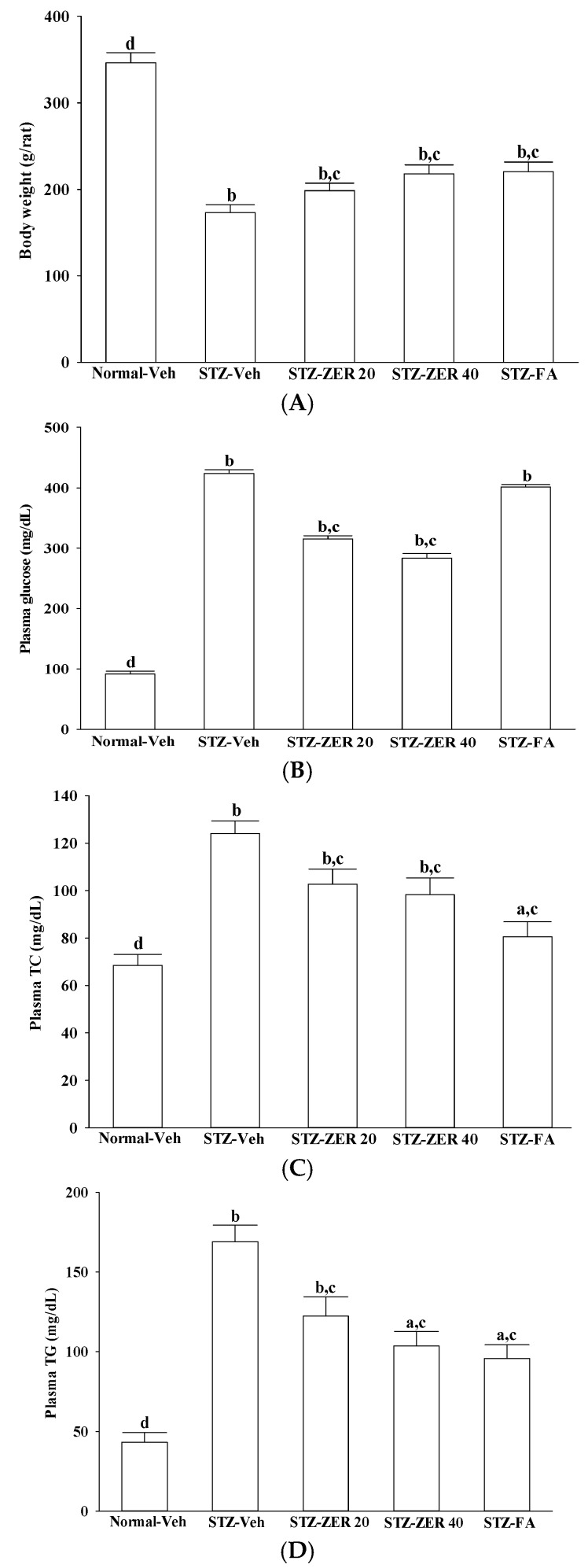
Changes in body weight (**A**) and plasma glucose (**B**), TC (**C**) and TG (**D**) in normal or STZ-diabetic rats receiving three months of treatment. STZ-diabetic rats were administered 20 mg/kg/day zerumbone (STZ-ZER 20), 40 mg/kg/day zerumbone (STZ-ZER 40) or 100 mg/kg fenofibric acid (STZ-FA) by oral gavage once daily for three months. Another group of STZ-diabetic rats (STZ-Veh) and normal rats (Normal-Veh) were administered the same volume of vehicle used to prepare the test medication solutions. Data are mean ± SD from eight rats per group. ^a^ *p* < 0.05 and ^b^ *p* < 0.01 compared to vehicle-treated normal rats, respectively; ^c^ *p* < 0.05 and ^d^ *p* < 0.01 compared to the values of vehicle-treated STZ-diabetic rats, respectively.

At the end of the experimental period, the body weights of the STZ-diabetic rats were lower than those of the normal rats (Figure 1). However, the body weights of zerumbone (40 mg/kg/day)-treated STZ-diabetic rats were 25.9% greater than their vehicle-treated counterparts. Similar results were seen in STZ-diabetic rats treated with fenofibric acid (100 mg/kg/day; Figure 1).

A significant increase in fasting plasma glucose in STZ-diabetic rats was observed compared with the normal group. Zerumbone at the oral dosage of 40 mg/kg/day reduced plasma glucose levels in STZ-diabetic rats to 66.9% that seen in their vehicle-treated counterparts (Figure 1). The plasma glucose levels in STZ-diabetic rats receiving fenofibric acid treatment were 5.2% lower than in their vehicle-treated counterparts, but the difference was not significant (Figure 1).

STZ-diabetic rats had significant increases in plasma TC and TG levels when compared with normal rats. The plasma TC and TG levels were reduced by 20.9% and 39.1%, respectively, in STZ-diabetic rats treated with 40 mg/kg/day zerumbone, compared with their vehicle-treated counterparts (Figure 1). Fenofibric acid treatment reduced plasma TC and TG levels in STZ-diabetic rats by 35.4% and 43.7% respectively, relative to the levels in vehicle-treated STZ-diabetic rats (Figure 1). No mortalities or clinical signs of toxicity were observed in STZ-diabetic rats receiving zerumbone or fenofibric acid treatment during the experimental period.

### 2.2. Fundus Photographs and Microvasculature Diameter

**Figure 2:**
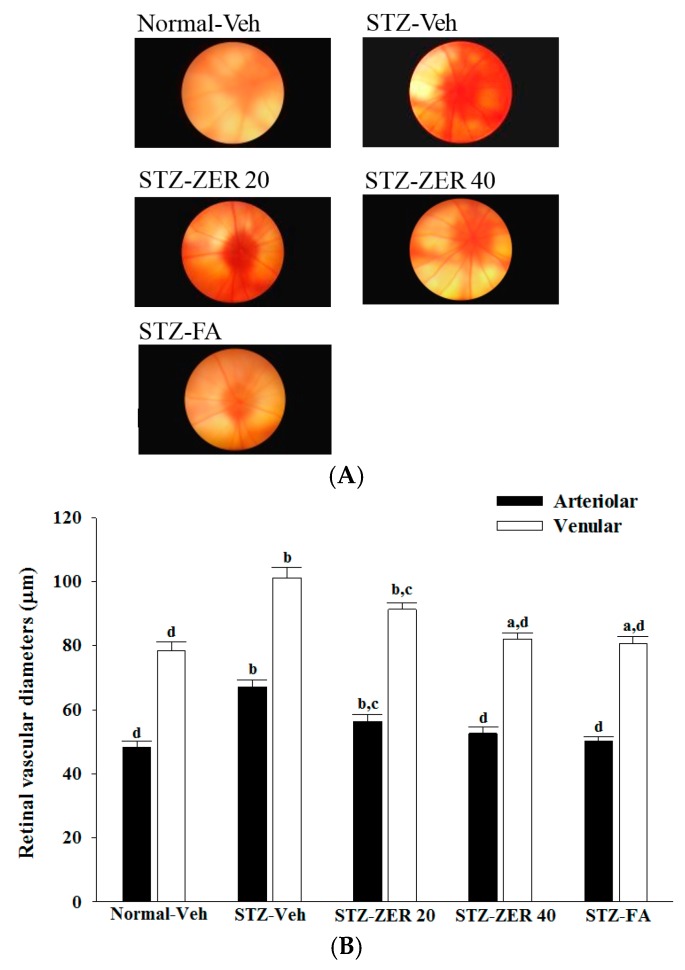
Fundus photographs (**A**) and changes in retinal vascular diameters (**B**) in normal or STZ-diabetic rats receiving three months of treatment. STZ-diabetic rats were administered 20 mg/kg/day zerumbone (STZ-ZER 20), 40 mg/kg/day zerumbone (STZ-ZER 40) or 100 mg/kg fenofibric acid (STZ-FA) by oral gavage once daily for three months. Another group of STZ-diabetic rats (STZ-Veh) and normal rats (Normal-Veh) were administered the same volume of vehicle used to prepare the test medication solutions. Data are mean ± SD from eight rats per group. ^a^ *p* < 0.05 and ^b^ *p* < 0.01 compared to vehicle-treated normal rats, respectively; ^c^ *p* < 0.05 and ^d^ *p* < 0.01 compared to the values of vehicle-treated STZ-diabetic rats, respectively.

Fundus photographs in the normal group show normal vascular architecture (Figure 2). Retinal arterioles and venules in STZ-diabetic rats were more dilated than those in the normal group, but the retinal vessels became mildly dilated in zerumbone (40 mg/kg/day)-treated STZ-diabetic rats (Figure 2). Similarly, fenofibric acid-treated retinae showed fewer dilated vessels (Figure 2).

### 2.3. Effects on Retinal Vascular Permeability

**Figure 3:**
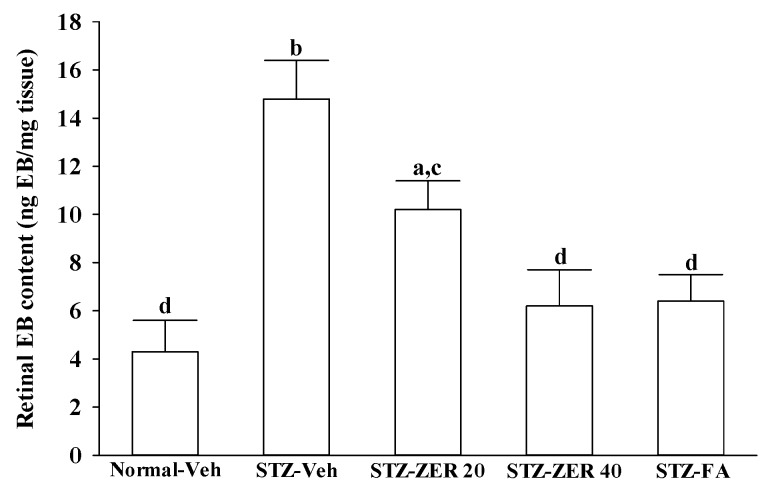
Effects on retinal vascular permeability. STZ-diabetic rats were administered 20 mg/kg/day zerumbone (STZ-ZER 20), 40 mg/kg/day zerumbone (STZ-ZER 40) or 100 mg/kg fenofibric acid (STZ-FA) by oral gavage once daily for three months. Another group of STZ-diabetic rats (STZ-Veh) and normal rats (Normal-Veh) were administered the same volume of vehicle used to prepare the test medication solutions. Retinal vascular permeability was measured with EB dye as a tracer. EB was normalized by total protein concentration in the tissue. Permeability is expressed as ng of dye per mg of protein in the tissue. Data are mean ± SD from eight rats per group, and the experiments were repeated independently at least three times with similar results. ^a^ *p* < 0.05 and ^b^ *p* < 0.01 compared to vehicle-treated normal rats, respectively; ^c^ *p* < 0.05 and ^d^ *p* < 0.01 compared to the values of vehicle-treated STZ-diabetic rats, respectively.

Increased leakage was observed in the retinas of STZ-diabetic rats (Figure 3). Treatment of STZ-diabetic rats with 20 or 40 mg/kg/day zerumbone for three months decreased retinal EB dye accumulation by 31.8% and 58.1%, respectively, when compared with the levels observed in the vehicle-treated counterparts (Figure 3). Diabetes-induced retinal vessel leakage was decreased by 56.7% in STZ-diabetic rats treated with fenofibric acid when compared with their vehicle-paired counterparts (Figure 3).

### 2.4. Effects on Retinal Leukostasis

**Figure 4:**
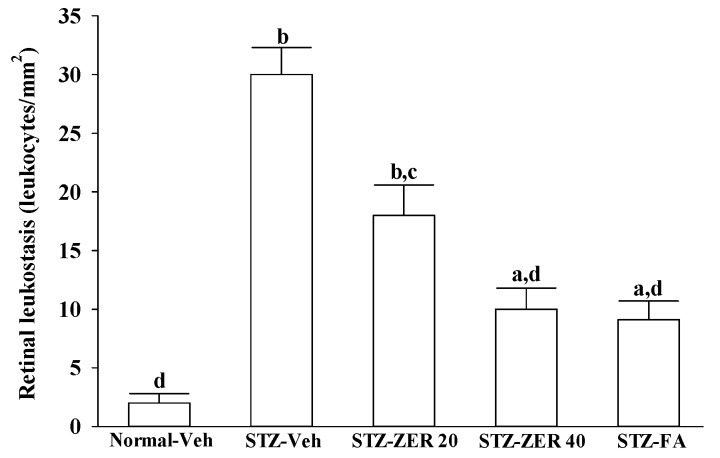
Effects on retinal leukostasis. STZ-diabetic rats were administered 20 mg/kg/day zerumbone (STZ-ZER 20), 40 mg/kg/day zerumbone (STZ-ZER 40) or 100 mg/kg fenofibric acid (STZ-FA) by oral gavage once daily for three months. Another group of STZ-diabetic rats (STZ-Veh) and normal rats (Normal-Veh) were administered the same volume of vehicle used to prepare the test medication solutions. The total number of adherent leukocytes per retina was counted. Data are mean ± SD from eight rats per group, and the experiments were repeated independently at least three times with similar results. ^a^ *p* < 0.05 and ^b^ *p* < 0.01 compared to vehicle-treated normal rats, respectively; ^c^ *p* < 0.05 and ^d^ *p* < 0.01 compared to the values of vehicle-treated STZ-diabetic rats, respectively.

Representative images of adherent leucocytes in retinal vessels from rats receiving three-months of treatments are shown in Figure 4. 

The number of adherent leukocytes in the retinal microvasculature of normal rats was negligible, while STZ-induced diabetes caused a significant increase in leukocytes adhering to the endothelia cells (Figure 4). Three-month administration of STZ-diabetic rats with zerumbone (40 mg/kg/day) significantly decreased retinal leukostasis by 66.7% compared to the vehicle-treated diabetic group (Figure 4). Treatment of STZ-diabetic rats with fenofibric acid lessens the diabetes-induced leukostasis to 30.3% of the level seen in their vehicle-treated counterparts (Figure 4).

### 2.5. Effects on Retinal Inflammatory Cytokines Expression

**Figure 5:**
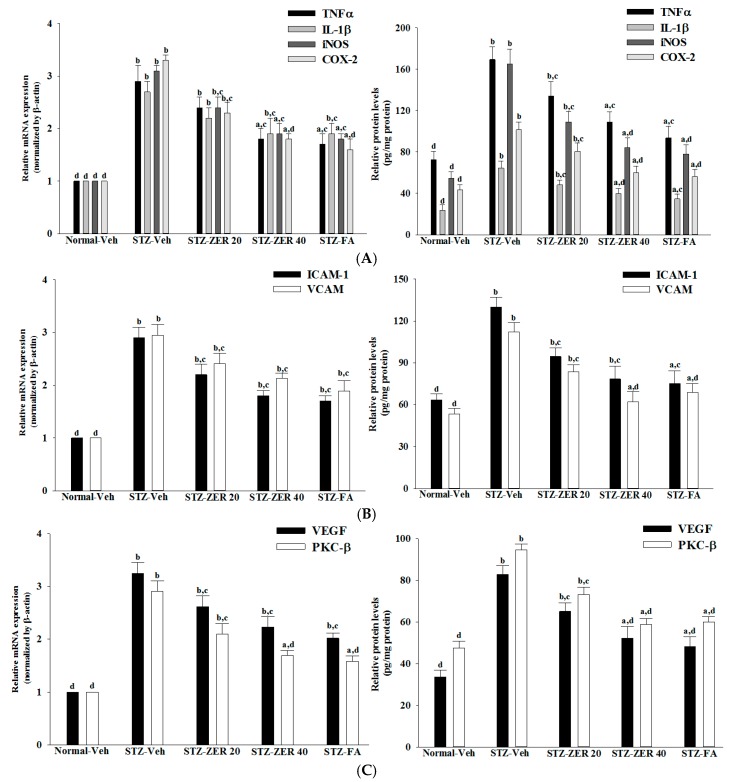
Effects on mRNA (left pannels) and protein expression (right pannels) of inflammatory cytokines (**A**), chemokines (**B**) and angiogenic parameters (**C**). STZ-diabetic rats were administered 20 mg/kg/day zerumbone (STZ-ZER 20), 40 mg/kg/day zerumbone (STZ-ZER 40) or 100 mg/kg fenofibric acid (STZ-FA) by oral gavage once daily for three months. Another group of STZ-diabetic rats (STZ-Veh) and normal rats (Normal-Veh) were administered the same volume. Data are mean ± SD from eight rats per group, and the experiments were repeated independently at least three times with similar results. ^a^ *p* < 0.05 and ^b^ *p* < 0.01 compared to vehicle-treated normal rats, respectively; ^c^ *p* < 0.05 and ^d^ *p* < 0.01 compared to the values of vehicle-treated STZ-diabetic rats, respectively.

STZ caused a 2.9-fold increase in retinal TNF-α mRNA, a 2.7-fold rise in retinal IL-1β mRNA, a 3.1-fold increase in retinal iNOS mRNA and a 3.3-fold rise in retinal COX-2 mRNA compared to the levels seen in the normal group (Figure 5A). The STZ-induced mRNA levels of TNF-α, IL-1β, iNOS and COX-2 in retinas were markedly reversed after 40 mg/kg/day zerumbone treatment (with decreases of 37.9%, 29.6%, 25.9% and 42.4%, respectively) compared to the levels seen in the vehicle-paired counterparts (Figure 5A). Fenofibric acid suppressed the STZ-induced stimulation of retinal mRNA levels of TNF-α, IL-1β, iNOS and COX-2 to 41.4%, 29.6%, 41.5% and 45.4%, respectively, compared to the levels seen in their vehicle-treated counterparts (Figure 5A).

In STZ-diabetic rats, the retinal protein levels of TNF-α, IL-1β, iNOS and COX-2 were significantly increased by around 1.7-, 2.7-, 3.0- and 2.3-fold, respectively, as compared to those seen in the normal group (Figure 5A). The retinal protein levels of TNF-α, IL-1β, iNOS and COX-2 in STZ-diabetic rats receiving 40 mg/kg/day zerumbone treatment were significantly decreased to 79.3%, 74.8%, 66.1% and 79.3% relative to the expression levels in vehicle-paired counterparts, respectively (Figure 5A). Giving STZ-diabetic rats fenofibric acid for three months significantly suppressed the retinal protein levels of TNF-α, IL-1β, iNOS and COX-2 to 69.7%, 54.5%, 47.1% and 55.3% relative to those seen in the vehicle-treated counterparts, respectively (Figure 5A).

### 2.6. Effects on Retinal Chemokines Expression

The retinal mRNA levels of ICAM-1 and VCAM-1 were 2.9- and 2.9-fold higher in STZ-diabetic rats than in the normal group, respectively, which were down-regulated by 40 mg/kg/day zerumbone treatment, with decreases of 45.1% and 37.9%, respectively, when compared with the levels observed in the vehicle-treated counterparts (Figure 5B). STZ-diabetic rats receiving fenofibric acid treatment had significantly lower retinal ICAM-1 and VCAM-1 mRNA levels of 34.4% and 34.8%, respectively, compared to their vehicle-treated counterparts (Figure 5B).

The protein levels of ICAM-1 and VCAM-1 in the retinas of STZ-diabetic rats were 2.0- and 2.1-fold higher than those of the normal group, respectively (Figure 5B). These STZ-induced up-regulations in protein levels of ICAM-1 and VCAM-1 were lower in the retina after treatment with 40 mg/kg/day zerumbone, at 60.3% and 55.4% the levels seen in the vehicle-treated STZ-diabetic rats, respectively (Figure 5B). Administration of fenofibric acid to STZ-diabetic rats significantly down-regulated the retinal ICAM-1 and VCAM-1 protein levels by 37.8% and 38.6%, respectively, relative to those in the vehicle-treated counterparts (Figure 5B).

### 2.7. Effects on Retinal Angiogenic Parameters Expression

The retinal expression of VEGF was significantly increased in the STZ-diabetic rats compared with the levels seen in normal rats at both mRNA and protein levels, which were decreased by 40 mg/kg/day zerumbone treatment to 67.7% and 63.1%, respectively, relative to those observed in the vehicle-treated counterparts (Figure 5C). Fenofibric acid treatment also decreased retinal VEGF mRNA and protein levels to 64.5% and 74.2%, respectively, of those in vehicle-treated STZ-diabetic rats (Figure 5C).

Retinal mRNA and protein levels of PKC-β in STZ-diabetic rats were clearly higher than those of the normal rats, and were down-regulated by 40 mg/kg/day zerumbone treatment, with decreases of 37.1% and 39.6%, respectively, when compared with the levels observed in the vehicle-treated counterparts (Figure 5C). Similar results were obtained in STZ-diabetic rats receiving fenofibric acid treatment (Figure 5C).

### 2.8. Effects on Retinal p38 MAPK Activation

**Figure 6:**
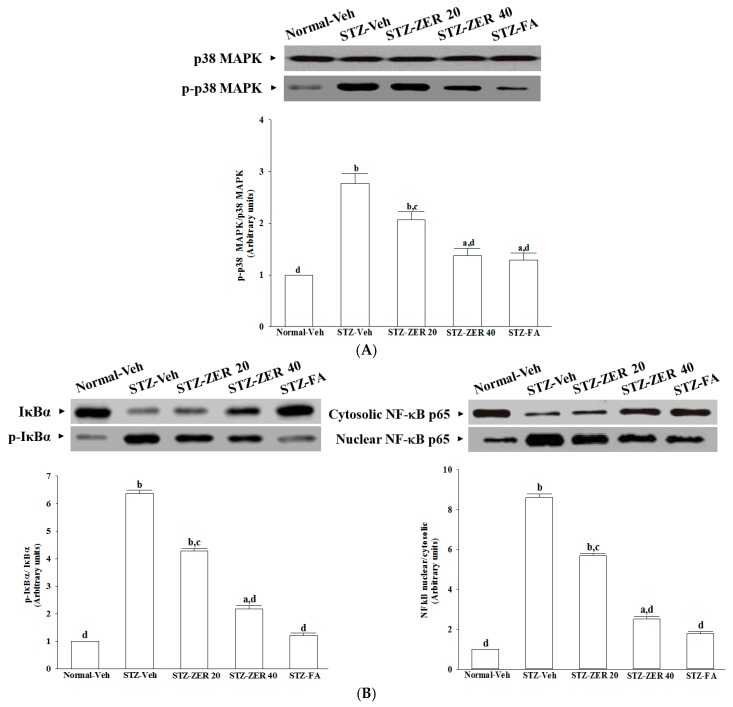
Effects on retinal p38 MAPK (**A**) and NF-κB (**B**) activation. STZ-diabetic rats were administered 20 mg/kg/day zerumbone (STZ-ZER 20), 40 mg/kg/day zerumbone (STZ-ZER 40) or 100 mg/kg fenofibric acid (STZ-FA) by oral gavage once daily for three months. Another group of STZ-diabetic rats (STZ-Veh) and normal rats (Normal-Veh) were administered the same volume. The expression and phosporylation of retinal IkB protein have been indicated in left pannel on Figure 6B. NF-κB p65 expression in nuclear and cytosolic fractions of retinas from rats has been shown in right pannel on Figure 6B. Data are mean ± SD from eight rats per group, and the experiments were repeated independently at least three times with similar results. ^a^ *p* < 0.05 and ^b^ *p* < 0.01 compared to vehicle-treated normal rats, respectively; ^c^ *p* < 0.05 and ^d^ *p* < 0.01 compared to the values of vehicle-treated STZ-diabetic rats, respectively.

The STZ significantly increased the p-p38 MAPK/p38 MAPK ratio in retinas of the rats (by 2.8-fold relative to that seen in the vehicle-treated normal rats) (Figure 6A). This STZ-induced up-regulation in the ratio of p-p38 MAPK/p38 MAPK was reversed in the retinas after treatment with 40 mg/kg/day, with a 49.5% decrease relative to that seen in vehicle-treated STZ-diabetic rats (Figure 6A). Treatment of STZ-diabetic rats with fenofibric acid also significantly down-regulated the ratio of p-p38 MAPK/p38 MAPK in the retinas to 46.5% relative to that seen in vehicle-treated STZ-diabetic rats (Figure 6A).

### 2.9. Effects on Retinal NF-κB Signaling Pathways

The phosphorylation levels of IκBα were 2.7-fold higher in the retinas of STZ-diabetic rats compared to those in the normal group (Figure 6B). The phosphorylation of IκBα in the retinas of STZ-diabetic rats were reduced by zerumbone treatment in a dose-dependent manner (Figure 6B). Similarly, fenofibric acid decreased the IκBα phosphorylation content in the retinas of STZ-diabetic rats to levels near those in normal rats (Figure 6B).

The nuclear-to-cytosolic protein expression ratio of NF-κB p65 protein was 8.6-fold higher in the retinas of STZ-diabetic rats compared to that in the normal group (Figure 6B). Zerumbone (40 mg/kg/day)-treated STZ-diabetic rats had 70.9% lower nuclear-to-cytosolic NF-κB p65 protein expression ratio in the retinas compared to that of their vehicle-treated counterparts (Figure 6B). Fenofibric acid suppressed the nuclear-to-cytosolic protein expression ratio of NF-κB p65 protein in the retinas of STZ-diabetic rats by 20.8% relative to that seen in their untreated counterparts (Figure 6B).

## 3. Materials and Methods

### 3.1. Experimental Animals

All experimental methods and animal care procedures were approved by the Institutional Animal Care and Use Committee (IACUC) of Tajen University (approval number, IACUC 104-10; approval date: 12 October 2015), in accordance with the Guide for the Care and Use of Laboratory Animals of the National Institutes of Health, as well as the guidelines of the Animal Welfare Act. Male Wistar rats weighting 200–250 g were purchased from the National Laboratory Animal Center (Taipei, Taiwan) and housed two per cage in a room under controlled temperature (20–25 °C), humidity (50% ± 5%) and lighting (12-h light/dark cycle) with food and water provided ad libitum. Rats were rendered diabetic by a single intravenous injection of 60 mg/kg streptozotocin (STZ; Sigma-Aldrich, Inc., St. Louis, MO, USA). Age-matched control rats were injected with vehicle (sterile saline 0.9%, pH 7.4). After one week, rats with non-fasting blood glucose levels >350 mg/dL, polyuria, and glucosuria were defined as diabetic and used for the experiments.

### 3.2. Treatment Protocols

At two weeks after the injection of STZ, a group of eight rats were dosed by oral gavage once per day for three consecutive months with zerumbone (≥98%; Sigma-Aldrich, Inc.) doses of 20 or 40 mg/kg in a volume of 1.5 mL/kg distilled water. The dosage regime was selected based on a previous report demonstrating that zerumbone at these dosages is potentially effective in improving diabetic nephropathy in STZ-diabetic rats [15]. Another group of STZ-diabetic rats was treated orally for three months with 100 mg/kg/day fenofibric acid (purity ≥98.0%; Sigma-Aldrich, Inc.), which was based on studies which reported this can have beneficial effects on DR rats [20]. A vehicle-treated group of normal rats and STZ-diabetic rats were treated with 1.5 mL/kg distilled water only over the same treatment period. Animals had free access to standard rat diet (Harlan Teklad, Madison, WI, USA; Cat. No. 2018) and water throughout the entire period.

At the end of the three-month treatment, the rats were weighed, fasted overnight and anesthetized using an intraperitoneal injection of sodium pentobarbital (60 mg/kg). While under anesthesia, they were painlessly sacrificed and blood was collected from the abdominal aorta of each animal into heparin sample bottles. Rat eyes from each group were removed and the retinae were isolated.

### 3.3. Biochemical Analysis

Blood samples were centrifuged at 2000× *g* for 10 min at 4 °C. The plasma was removed and placed into aliquots for analyses. Kits for determining plasma levels of glucose (Cat. No. 10009582), total cholesterol (TC; Cat. # 10007640) and triglyceride (TG; Cat. # 1001030) were purchased from Cayman Chemical Company, Inc. (Ann Arbor, MI, USA). All experimental assays were carried out according to the manufacturers’ instructions; all samples were analyzed in triplicate.

### 3.4. Fundus Photography and Vessel Diameter

Fundus photography was performed with a retina camera (Kowa Company Ltd., Tokyo, Japan). In order to accustom the rats to the fundus photography procedure, the animals were trained before the start of the study. Eyes were dilated with a drop of 1% tropicamide (Synpac-Kingdom Pharmaceutical Co., Ltd., Taipei, Taiwan). Moisol eye drops (0.7% HPMC) were administered periodically to avoid drying of the cornea. Fundus photography was done regularly till three months to monitor the fundus changes.

The diameter of retinal vessels was estimated by a previously described method [21]. Before diameter estimation, the retinal photographs from all groups were randomized. The vessel diameters of the three most prominent vessels were estimated at three sites in its widest portion at an equal distance from the center. An average of three estimations was taken as the final retinal vessel diameter.

### 3.5. Quantification of Retinal Leukostasis

Quantification of leukostasis was performed at the end of the three-month treatment by a previously described method [22]. Rats were anesthetized with sodium pentobarbital (40 mg/kg body weight), then a 20-gauge perfusion needle was inserted through the left ventricle into the base of the aortic arch, making sure that the needle did not obstruct the carotid arteries. The needle was clamped in place, then the right atrium was cut to allow outflow. Rats were perfused with 10 mL of phosphate buffered saline (PBS) and heparin (0.1 mg/mL) to wash out nonadherent blood cells. Fluorescein isothiocyanate-coupled concanavalin A lectin (Con A) (20 μg/mL in PBS; pH 7.4; 5 mg/kg body weight; Vector Laboratories, Burlingame, CA, USA) was then perfused to label adherent leukocytes and vascular endothelial cells. Unbound ConA was flushed by perfusion with 10 mL PBS. Eyes were removed and fixed in 4% paraformaldehyde for 1 h. Retinas were dissected and flat mounted on a microscope slide, covered with anti-fading medium and a coverslip, and imaged via fluorescence microscopy. Only whole retinas in which the entire vascular network was stained were used for analysis. The total number of adherent leukocytes within the vessels of each retina was counted.

### 3.6. Measurement of Retinal Vascular Permeability

Retinal vascular permeability was measured using the Evans blue (EB) dye extravasation technique at the end of the three-month treatment [23]. EB dye (Sigma-Aldrich, Inc.) was dissolved in normal saline at 45 mg/mL and was injected through the tail vein of anesthetized rats over 10 s at a dosage of 45 mg/kg. After the dye had circulated for two hours, the rats were anesthetized with sodium pentobarbital (40 mg/kg body weight), the chest cavity was opened, and cardiac perfusion was performed via the left ventricle with 1% paraformaldehyde in citrate buffer (0.05 mol/L, pH 3.5) under a constant pressure of 120 mmHg. Immediately after perfusion, the retinas were carefully dissected under an operating microscope. After the retinas were fully dried at 4 °C, their weights were measured and the EB dye was extracted by incubating each sample in 150 µL formamide for 18 h at 70 °C. The extract was ultracentrifuged at a speed of 14,000 rpm for 60 min. Absorbance was measured using 100 µL of the supernatant at 620 nm and 740 nm. The concentration of EB in the extracts was calculated from a standard curve and normalized by the weight of the dry retina.

### 3.7. Retinal Inflammatory and Angiogenic Parameters Determination

Retinas were lysed in ice-cold radio-immunoprecipitation assay buffer (50 mmol/L, Tris-HCl (pH 8), 150 mmol/L NaCl, 1 mmol/L ethylenediaminetetraacetic acid (EDTA), 0.1% sodium dodecyl sulfate, 1% IGEPAL^®^ CA-630 (Sigma-Aldrich, Inc.) and 0.5% sodium deoxicholate) containing protease inhibitor cocktails (Sigma-Aldrich, Inc.; Cat. No. P83490) and centrifuged for 15 min at 10,000× *g* at 4 °C [24]. The supernatants were collected and assayed for protein content using a Bio-Rad protein assay kit (Bio-Rad Laboratories, Milan, Italy). Enzyme-linked immunosorbent assay (ELISA) kits for the determination of TNF-α (Cat. No. ab46070), IL-1β (Cat. No. ab100768) and intercellular adhesion molecule-1 (ICAM-1; Cat. No. ab100763) were obtained from Abcam Inc. (Cambridge, MA, USA). Vascular cell adhesion molecule 1 (VCAM-1) ELISA kit (Cat. No. NB-E30582) was obtained from Novatein Biosciences (Cambridge, MA, USA). Rat iNOS (Cat. No. E-EL-R0520), COX-2 (Cat. No. E-EL-R0792), VEGF (Cat. No. E-EL-R0020) and protein kinase C (PKC)-β1 (Cat. No. E-EL-R0808) ELISA kits were purchased from Elabscience Biotechnology Co., Ltd. (Wuhan, Hubei, China). All assays were carried out in triplicate.

### 3.8. Preparation of Nuclear and Cytoplasmic Fractions

Retinas were homogenized in a western lysis buffer (30 mmol/L Tris-HCl, pH 7.4, 250 mmol/L Na_3_VO_4_, 5 mmol/L EDTA, 250 mmol/L sucrose, 1% Triton X-100 with protease inhibitor and phosphatase inhibitor cocktail). The homogenate was centrifuged at 800× *g* for 5 min at 4 °C, and supernatant containing the cytosolic extract was stored frozen at –80 °C. The nuclear pellet was resuspended in 25 µL ice-cold nuclear extraction buffer (20 mmol/L 4-(2-hydroxyethyl)-1-piperazineethanesulfonic acid, 0.4 mmol/L NaCl, 1 mmol/L EDTA, 25% glycerol, protease inhibitors 1X). After 30 min of intermittent mixing, the extract was centrifuged at 14,000× *g* for 10 min at 4 °C, and supernatants containing nuclear extracts were secured.

### 3.9. Western Blot Analysis

Before immunoblotting, the protein concentration of each tissue was determined using a Bio-Rad protein assay kit (Bio-Rad Laboratories, Tokyo, Japan), with bovine serum albumin as a standard to ensure equal loading among lanes. Cytosolic (70 μg total protein) and nuclear (50 μg total protein) extracts were separated on a 7.5%–15% polyacrilamide gel and transferred electophoretically to nitrocellulose membranes. Membranes were blocked with 5% non-fat dry milk in Tris-buffered saline Tween (20 mmol/L Tris, pH 7.6, 137 mmol/L NaCl, and 0.1% Tween 20) for three hours at room temperature and incubated overnight at 4 °C with primary antibodies against p38 mitogen-activated protein kinase (p38 MAPK, Cell Signaling Technology, Beverly, MA, USA; Cat. No. 9212), phospho-p38 MAPK (Thr180/Tyr182) (p-p38 MAPK, Cell Signaling Technology; Cat. No. 9211), inhibitory kappa B (IκBα, Santa Cruz Biotechnology, Inc., Santa Cruz, CA, USA; Cat. No. sc-371) and NF-κB p65 (Santa Cruz Biotechnology, Inc.; Cat. No. sc-109). All antibodies were used at a dilution of 1:1000. After washing three times with Tris-buffered saline with Tween 20 (TBST), the membranes were labeled with horseradish peroxidase-conjugated secondary antibodies for one hour at room temperature. After three additional washes with TBST, the immunoreactive bands were visualized using an enhanced chemiluminescence system (Amersham Biosciences, Buckinghamshire, UK) according to the manufacturer’s instructions. Films were scanned, and band densities were quantified with densitometric analysis using ATTO Densitograph Software (ATTO Corp., Tokyo, Japan). All values were normalized by setting the density of the control samples as 1.0. Tissue sections were sampled for four independent experiments.

### 3.10. Real-Time Polymerase Chain Reaction (PCR)

Total RNA was extracted from rat retinas using a Trizol reagent (Invitrogen; Boston, MA, USA) according to the manufacturer’s protocol. RNA was quantified by A260 and its integrity verified by agarose gel electrophoresis using ethidium bromide for visualization. For the reverse transcriptase reaction, 1 μg of total RNA per sample and 8.5 μg/μL random hexamer primers were heated at 65 °C for 5 min and then quenched on ice. This mixture was combined with 500 μmol/L each of dATP, dTTP, dCTP, and dGTP, 10 mmol/L DTT, 20 mmol/L Tris-HCl (pH 8.4), 50 mmol/L KCl, 5 mmol/L MgCl_2_, 40 units of RNaseOUT^TM^ recombinant ribonuclease inhibitor (Invitrogen) and 100 units SuperScript III reverse transcriptase (Invitrogen). Samples were subjected to DNase (Promega; Madison, WI, USA) treatment at 37 °C for 20 min in a GeneAmp 9700 Thermal Cycler (Applied Biosystems; Foster City, CA, USA) and then held at 4 °C. After aliquots were taken for immediate use in PCR, the remainder of the cDNA was stored at −20 °C. mRNA expression was measured by quantitative real-time PCR in a fluorescent temperature Lightcycler 480 (Roche Diagnostics; Mannheim, Germany). The following primer sequences were used: 5′-ACACCATGAGCACGGAAA GC-3′ (forward) and 5′-CCGCCACGAGCAGGAA-3′ (reverse) for TNF-𝛼; 5′-AATGGACAGAACAT AAGCCAACA-3′ (forward) and 5′-CCCAAGGCCACAGGGAT-3′ (reverse) for IL-1β; 5′-TGATCTT GTGCTGGAGGTGACCAT-3′ (forward) and 5′-TGTAGCGCTGTGTGTCACAGAAGT-3′ (reverse) for iNOS; 5′-GCATTCTTTGCCCAGCACTTCACT-3′ (forward) and 5′-TTTAAGTCCACTCCATGGCCCA GT-3′ (reverse) for COX-2; 5′-CGGGTTTGGGCTTCTCC-3′ (forward) and 5′-GCCACTGCTCGTCCAC ATAG-3′ (reverse) for ICAM-1; 5′-ATCTTCGGAGCCTCAACGG-3′ (forward) and 5′-CCAATCTGAG CGAGCGTTT-3′ (reverse) for VCAM-1; 5′-ACAGGGAAGACAATG GGATGA-3′ (forward) and 5′-GGGCCAGGGATGGGTTT-3′ (reverse) for VEGF; 5′-ACGAATTTGCTGGCTTCTCC-3′ (forward) and 5′-TGGCCTGAAGTCTTACACTCCA-3′ (reverse) for PKC-β; and 5′-TGTGATGGTGGGAATGGGTC AG-3′ (forward) and 5′-TTTGATGTCACGCACGATTTCC-3′ (reverse) for β-actin. Primers were designed using Primer Express Software version 2.0 System (Applied Biosystems; Foster City, CA, USA). The PCR reaction was performed using the following cycling protocol: 95 °C for 5 min, followed by 45 cycles of 95 °C for 5 s, 58 °C for 15 s, and 72 °C for 20 s. Dissociation curves were run after amplification to identify the specific PCR products. The mRNA expression levels were normalized to β-actin mRNA levels and calculated according to the delta-delta Ct method [25].

### 3.11. Statistical Analysis

Data are expressed as the mean ± SEM. Statistical analysis and graphics were performed with the SigmaPlot 12.3 program (version 2016, Systat Software Inc., San Jose, CA, USA). Statistical analysis was performed with one-way analysis of variance (ANOVA). Dunnett range post-hoc comparisons were used to determine the source of significant differences, where appropriate. A *p* value of less than 0.05 was considered statistically significant.

## 4. Discussion

There is growing evidence that leukocyte adhesion to the retinal vasculature or retinal leukostasis results in BRB breakdown in early DR [26,27]. We observed that the number of leukocytes adhering to the retinal vascular endothelium was increased in STZ-diabetic rats, and that vascular permeability and the thickness of retinal vessels increased dramatically. In agreement with the attenuated leukostasis, zerumbone reduced retinal permeability accompanied by decreased vascular permeability of STZ-diabetic rats. The suppressive effect of zerumbone on the BRB breakdown may be important for the prevention of the progression of DR.

Retinal vascular hyperpermeability causes macular edema, leading to visual deterioration in retinal diseases such as DR [28]. VEGF, an angiogenic cytokine, is known to be a key molecule leading to retinal permeability and breakdown of BRB in diabetes and other retinal diseases [28]. VEGF-induced retinal permeability is in part through activation of PKC, specifically the β isoform [29]. We found that the elevated contents of VEGF and PKC-β in retinas of STZ-diabetic rats were both reduced in rats receiving zerumbone treatment. It is thus suggested that zerumbone has a protective effect on diabetes induced vasculopathy via the repression of vascular hyperpermability.

Extensive research has verified the potential role of inflammatory mediators in DR [5]. One of these mediators is TNF-α, a proinflammatory cytokine which is known as an initiator of inflammatory reactions [30]. Similarly, IL-1β, iNOS and COX-2 can be up-regulated in the retina with diabetes [31,32]. In addition to increases in the above-mentioned inflammatory mediators, ICAM-1 and VCAM-1 promote chemoattraction of leukocytes into the vascular walls and their migration into retinal tissues [33]. Zerumbone have been demonstrated to attenuate the expression of proinflammatory factors, chemokines, or adhesion molecules in the kidneys of STZ-diabetic rats [15]. Notably, we found that zerumbone down-regulated the gene expression of a series of proinflammatory cytokines, which may consequently act directly in the retina to reduce the expression of chemokines and adhesion molecules, finally leading to reduced leukocytosis in the retinas of STZ-diabetic rats. These results support the notion that the protective effects of zerumbone with regard to retinal damage in STZ-diabetic rats may be via blockade of inflammation and inhibition of leukostasis/monocyte adhesion to the capillary endothelium [16].

The increase in leukostasis was also associated with the activation of NF-κB, an important transcription factor involved in inflammatory responses [7]. Once activated by inflammatory mediators, NF-κB will be dissociated from IκBα and translocate into the nucleus to modulate the transcription of its target genes [33]. NF-κB activation is known to induce the adhesion molecules, cytokines, growth factors and iNOS expression [34]. NF-κB inhibition has been found to reduce the levels of leukostasis and BRB breakdown in diabetic rat retinas [31]. Zerumbone has been reported to exert anti-inflammatory activity through inhibiting NF-κB activation in mouse cornea from UVB-induced photokeratitis [35]. The inhibition of NF-κB by zerumbone might be a critical step in preventing a cascading inflammatory response during progression of DR, as documented in our previous study [16]. The current work provides results which suggest that zerumbone inhibits activation of the NF-κB pathway via blockade of the degradation and phosphorylation of IκB.

In addition to NF-κB, the activation of p38 MAPK has been reported in the retinas of diabetic rats, and is associated with BRB breakdown [36]. In fact, the activation of p38 MAPK signaling contributes as an upstream regulator to stimulate the transcriptional activity of NF-κB [37]. We found that zerumbone decreased p38 MAPK activation in diabetic retinas, and this was accompanied by the suppression of NF-κB nuclear translocation, thus suggesting that the protective effects of zerumbone on DR-related inflammatory responses appear to be due to the inhibition of p38 MAPK-NF-κB-dependent pathways. Zerumbone possesses retinal protective effects, which might be associated with the blockade of the AGEs/RAGE pathway and result in downregulation of NF-κB-mediated inflammatory signals, as documented in our previous study [15]. It is thus likely that zerumbone has protective effects on several pharmacological targets in DR.

Nevertheless, as we mentioned previously, DR has a complicated etiology and involves many factors. Among these causative factors, dyslipidemia seems to contribute heavily [17]. In fact, zerumbone has been shown to ameliorate dyslipidemia in high-fat diet-induced hyperlipidemic hamsters through the enhancement of gene expression involved in the lipid metabolism through PPARα activation [18]. Our results also show that, along with the plasma glucose lowering action, zerumbone treatment significantly decreases the higher TG or TC levels in the plasma of STZ-diabetic rats. However, fenofibric acid ameliorates retinal damage with less glycemic control, in comparison with zerumbone. We thus propose that the beneficial effects of zerumbone on DR are at least in part through its anti-hyperglycemic and anti-hyperlipidemic actions. The retinal protective impact of zerumbone is as effective as that produced by fenofibric acid. As such, the role of glucose or lipid homeostasis with regard to the action of zerumbone on the amelioration of diabetes-associated retinal dysfunction is of considerable interest, and should be further clarified in future research.

## 5. Conclusions

Our results show that zerumbone has a protective effect on STZ induced DR in rats with possible mechanisms of inhibiting angiogenic activity and leukocytosis, ultimately acting against the retinal BRB breakdown. Impairment of p38 MAPK phosphorylation and NF-κB activation, thus limiting the inflammatory response, has also been suggested as a possible underlying mechanism of zerumbone with regard to preventing the progression of diabetic retinal vascular diseases. The anti-hyperglycemic and anti-hyperlipidemic effects of zerumbone may correlate with the reduction of the risk of DR. These findings contribute to a significantly better understanding of the beneficial effects of zerumbone with regard to diabetes, and can thus serve as the basis for the further therapeutic development of zerumbone in treating DR in future work.
